# Effective Activation of BK_Ca_ Channels by QO-40 (5-(Chloromethyl)-3-(Naphthalen-1-yl)-2-(Trifluoromethyl)Pyrazolo [1,5-a]pyrimidin-7(4*H*)-one), Known to Be an Opener of KCNQ2/Q3 Channels

**DOI:** 10.3390/ph14050388

**Published:** 2021-04-21

**Authors:** Wei-Ting Chang, Sheng-Nan Wu

**Affiliations:** 1 Institute of Clinical Medicine, College of Medicine, National Cheng Kung University, Tainan 70101, Taiwan; cmcvecho2@gmail.com; 2 Division of Cardiovascular Medicine, Chi-Mei Medical Center, Tainan 71004, Taiwan; 3 Department of Biotechnology, Southern Taiwan University of Science and Technology, Tainan 71004, Taiwan; 4 Department of Physiology, National Cheng Kung University Medical College, Tainan 70101, Taiwan; 5 Institute of Basic Medical Sciences, National Cheng Kung University Medical College, Tainan 70101, Taiwan; 6 Department of Medical Research, China Medical University Hospital, China Medical University, Taichung 40402, Taiwan

**Keywords:** OQ-40 (5-(chloromethyl)-3-(naphthalene-1-yl)-2-(trifluoromethyl)pyrazolo[1,5-a]pyrimidin-7-(4*H*)-one), Ca^2+^-activated K^+^ current, large-conductance Ca^2+^-activated K^+^ channel, single-channel kinetics, hysteresis

## Abstract

QO-40 (5-(chloromethyl)-3-(naphthalene-1-yl)-2-(trifluoromethyl) pyrazolo[1,5-a]pyrimidin-7(4*H*)-one) is a novel and selective activator of KCNQ2/KCNQ3 K^+^ channels. However, it remains largely unknown whether this compound can modify any other type of plasmalemmal ionic channel. The effects of QO-40 on ion channels in pituitary GH_3_ lactotrophs were investigated in this study. QO-40 stimulated Ca^2+^-activated K^+^ current (*I*_K(Ca)_) with an EC_50_ value of 2.3 μM in these cells. QO-40-stimulated *I*_K(Ca)_ was attenuated by the further addition of GAL-021 or paxilline but not by linopirdine or TRAM-34. In inside-out mode, this compound added to the intracellular leaflet of the detached patches stimulated large-conductance Ca^2+^-activated K^+^ (BK_Ca_) channels with no change in single-channel conductance; however, there was a decrease in the slow component of the mean closed time of BK_Ca_ channels. The *K*_D_ value required for the QO-40-mediated decrease in the slow component at the mean closure time was 1.96 μM. This compound shifted the steady-state activation curve of BK_Ca_ channels to a less positive voltage and decreased the gating charge of the channel. The application of QO-40 also increased the hysteretic strength of BK_Ca_ channels elicited by a long-lasting isosceles-triangular ramp voltage. In HEK293T cells expressing *α-hSlo*, QO-40 stimulated BK_Ca_ channel activity. Overall, these findings demonstrate that QO-40 can interact directly with the BK_Ca_ channel to increase the amplitude of *I*_K(Ca)_ in GH_3_ cells.

## 1. Introduction

**Figure 1:**
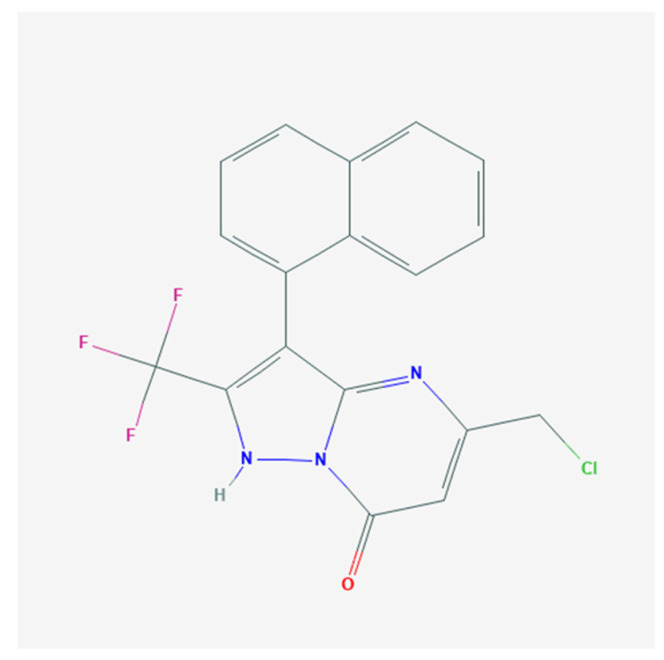
Chemical structure of QO-40.

QO-40 (5-(chloromethyl)-3-(naphthalene-1-yl)-2-(trifluoromethyl)pyrazolo[1,5-a]pyrimidin-7-(4*H*)-one) is a highly pure, synthetic, and biologically active compound (Figure 1). This compound has been previously reported to enhance KCNQ2/KCNQ3 heteromeric currents expressed in *Xenopus* oocytes [1]. QO58-lysine, a compound structurally similar to QO-40, can also activate neuronal KCNQ channels and exert antinociceptive effects on inflammatory pain [2,3,4,5,6]. The QO-58-induced amelioration of inflammatory pain observed in rodents was previously viewed as being accompanied by the activation of KCNQ-encoded K^+^ currents [3]. To date, however, none of the studies have thoroughly investigated with the underlying mechanism of action of QO-40 or its structurally similar compounds on other types of ionic currents (e.g., Ca^2+^-activated K^+^ current, [*I*_K(Ca)_]).

Big-, large-, or high-conductance Ca^2+^-activated K^+^ (BK_Ca_ or BK) channels (KCa1.1, KCNMA1, *Slo1*), which belong to a family of voltage-activated K^+^ channels, are stimulated by increasing cytosolic Ca^2+^ concentrations, membrane depolarization, or both. The channel activation of their own accord can conduct large amounts of K^+^ ions across the cell membrane. As a result of its high conductance, the BK_Ca_ channel is also thought to be a maxi- or large-K channel. These channels, which are functionally expressed in a wide variety of excitable or nonexcitable cells, can play a role in numerous physiological or pathological events, including neuronal excitability, neurotransmitter release, stimulus-secretion coupling, muscle relaxation, and pain sensation [7,8,9,10,11]. Some small synthetic or natural molecules have been previously reported to be regulators of BK_Ca_ channel activity [9,11,12]. BMS-204352 (MaxiPost^TM^), known to exert anxiolytic effects, can activate BK_Ca_ channels and KCNQ-encoded currents [13,14]. Similarly, naringenin, a bioflavonoid, reportedly activates the M-type K^+^ current (*I*_K(M)_) and the activity of BK_Ca_ channels [15].

Therefore, based on the above-stated initiatives, the aims of the present work were (1) to test the hypothesis that QO-40 can affect whole-cell Ca^2+^-activated (or Ca^2+^-dependent) K^+^ currents (*I*_K(Ca)_) in pituitary GH_3_ lactotrophs and (2) to address the issue of whether and how this compound can perturb the activity and kinetic properties of BK_Ca_ channels. The activity of BK_Ca_ channels has been previously demonstrated to be enriched in these cells [7,12]. In *α-hSlo*-expressing HEK293T cells, QO-40 also effectively stimulated BK_Ca_ cells. Our results provide the first evidence demonstrating that this compound can interact with the BK_Ca_ channel to increase the amplitude of *I*_K(Ca)_. Experiments described in this study are pertinently useful, as they highlight the potential mechanism of ionic actions of QO-40 or other structurally similar synthesized compounds (i.e., a series of pyrazolol[1,5-a]pyrimidin-7(4H)-one [PPO] derivatives), although these compounds have been used as activators of M-type K^+^ currents (*I*_K(M)_) [1,3,16].

## 2. Results

### 2.1. Stimulatory Effect of QO-40 on I_K(Ca)_ Recorded from Pituitary GH_3_ Lactotrophs

**Figure 2:**
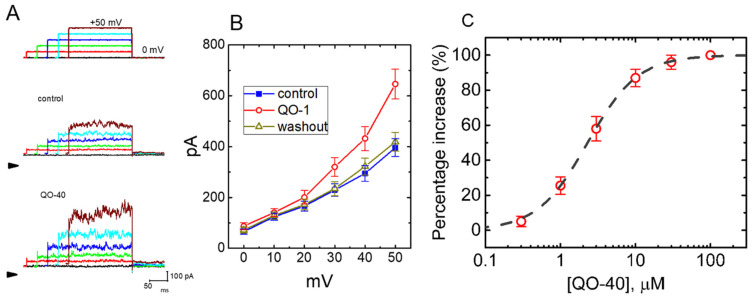
Stimulatory effect of QO-40 on the magnitude of whole-cell Ca^2+^-activated K^+^ current (*I*_K(Ca)_) recorded from GH_3_ pituitary tumor cells. This set of voltage-clamp experiments was undertaken in cells which were kept immersed in normal Tyrode’s solution containing 1.8 mM CaCl_2_; the recording pipet used was backfilled with a K^+^-containing solution. We elicited *I*_K(Ca)_ from a holding potential of 0 mV to test potentials in the range of 0 and +60 mV (10 mV increments) at a rate of 0.1 Hz. (**A**) Representative *I*_K(Ca)_ traces activated in response to a series of voltage steps (indicated in the uppermost part). Current traces in the upper part are controls (i.e., QO-40 was not present), while those in the lower part were obtained in the presence of 3 μM QO-40. Arrowhead in each panel denotes the zero-current level, calibration mark in the right lower corner applies to all current traces illustrated, and the duration of square voltage command pulse applied was set in the range of 300 and 180 ms (30 ms decrements). (**B**) Mean current–voltage (*I-V*) relationship of *I*_K(Ca)_ obtained in the control, during the exposure to 3 μM QO-40, or after washout of QO-40. Each point represents the mean SEM (*n* = 7–9). The statistical analyses were undertaken by ANOVA-2 for repeated measures, *p* (factor 1, groups among data taken at different levels of voltage) < 0.05, *p* (factor 2, groups between the absence and presence of 3 μM QO-40) < 0.05, *p* (interaction) < 0.05, followed by post hoc Fisher’s least-significant difference test, *p* < 0.05. (**C**) Concentration–response relationship for QO-40-induced stimulation of *I*_K(Ca)_. Current amplitude was taken at the end of depolarizing pulse from 0 to +50 mV. Data analysis was performed by ANOVA-1 (*p* < 0.05). The smooth dashed line is fitted to the Hill equation. The values for EC_50_, maximal percentage increase in *I*_K(Ca)_ amplitude, and Hill coefficient were yielded to be 2.3 μM, 100%, and 1.3, respectively. Each point represents the mean ± SEM (*n* = 8).

In the first stage of electrophysiological measurements, we performed the whole-cell configuration of the patch-clamp experiments to evaluate any possible adjustments of QO-40, a synthetic and biologically active compound, on *I*_K(Ca)_ identified from GH_3_ cells. We performed voltage-clamp current recordings as cells were immersed in HEPES-buffered normal Tyrode’s solution in which 1.8 mM CaCl_2_ was present, and the recording pipet was backfilled with a K^+^-containing solution that contained 140 mM K^+^, 0.1 mM EGTA and 3 mM ATP. As the whole-cell mode (i.e., in situations where the membrane patch was broken by suction) was achieved, we held the cell in the voltage clamp at 0 mV to ensure the inactivation of other types of outwardly rectifying K^+^ currents and minimal interference by voltage-activated Ca^2+^ currents [17,18,19]. Ionic currents were thereafter elicited in response to a series of voltage commands ranging between 0 and +60 mV. This type of macroscopic outward K^+^ current, which is biophysically characterized by a markedly noisy and outwardly rectifying property, is *I*_K(Ca)_ [7,17,20]. These currents are particularly subject to inhibition by tremorgenic mycotoxins (e.g., paxilline, penitrem A, or verruculogen) [21]. As demonstrated in Figure 2A,B, within 1 min of exposing GH_3_ to QO-40 (3 μM), there was a progressive increase in the *I*_K(Ca)_ amplitude measured throughout all voltage-clamp steps examined.

A concentration-dependent relationship of the QO-40-mediated stimulation of *I*_K(Ca)_ amplitude observed in GH_3_ cells is illustrated in Figure 2C. Of note, this compound led to the simulation of *I*_K(Ca)_ amplitude in a concentration-dependent manner. Based on a least-squares fit to the modified Hill equation, the experimental results yielded a concentration required for half-maximal stimulation (i.e., EC_50_) of 2.3 μM and a Hill coefficient of 1.3.

### 2.2. Comparisons among the Effects of QO-40, QO-40 Plus Linopirdine, QO-40 Plus TRAM-34, QO-40 Plus GAL-021 and QO-40 Plus Paxilline on I_K(Ca)_ Amplitude in GH_3_ Cells

**Figure 3:**
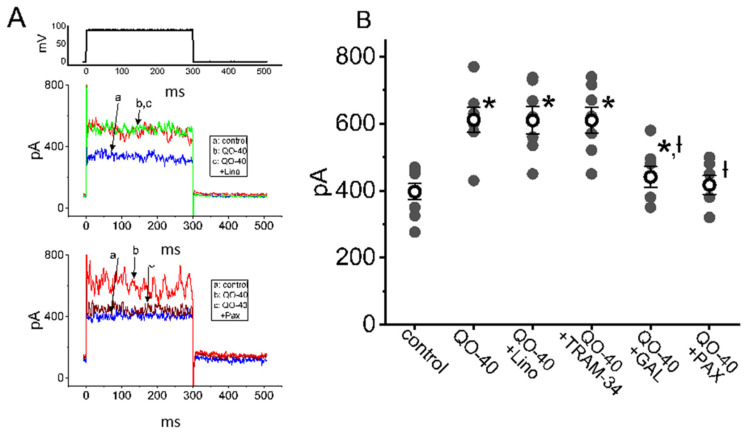
Effects of linopirdine, TRAM-34, GAL-021, and paxilline on QO-40-stimulated *I*_K(Ca)_ in GH_3_ cells. In this set of experiments, whole-cell current recordings were undertaken in cells bathed in normal Tyrode’s solution, and the pipet was backfilled with K^+^-containing internal solution. (**A**) Representative *I*_K(Ca)_ traces in the absence (a, blue color) and presence of either QO-40 (b, red color), QO-40 plus linopirdine (c, upper panel, green color), or QO-40 plus paxilline (c, lower panel, brown color). The uppermost part shows the voltage-clamp protocol used. (**B**) Vertical scatter graph showing effects of linopirdine, TRAM-34, GAL-021, or paxilline on QO-40-induced stimulation of *I*_K(Ca)_ (mean ± SEM; *n* = 6–8 for each point). QO-40: 3 μM QO-40; Lino: 10 μM linopirdine; TRAM-34: 3 μM TRAM-34; GAL-021: 3 μM GAL-021; Pax: 1 μM paxilline. Data analysis was performed by ANOVA-1 (*p* < 0.05). * Significantly different from control (*p* < 0.05) and † significantly different from QO-40 (3 μM) alone group (*p* < 0.05).

We next tested whether the stimulatory effect of QO-40 on *I*_K(Ca)_ was attenuated by linopirdine, TRAM-34, GAL-021, or paxilline. Linopirdine inhibits M-type K^+^ current (*I*_K(M)_), and TRAM-34 inhibits intermediate-conductance Ca^2+^-activated K^+^ (IK_Ca_) channels [15,22,23], while GAL-021 and paxilline suppress the activity of large-conductance Ca^2+^-activated K^+^ (BK_Ca_) channels [21,24,25]. As demonstrated in Figure 3, when cells were continually exposed to QO-40 (3 μM), neither linopirdine (10 μM) nor TRAM-34 (3 μM) could attenuate QO-40-stimulated *I*_K(Ca)_; conversely, the subsequent addition of GAL-021 (3 μM) or paxilline (1 μM) led to effective attenuation of the increase in *I*_K(Ca)_ amplitude. These findings demonstrated that the *I*_K(Ca)_ amplitude stimulated by QO-40 is not modified by blockers of *I*_K(M)_ or IK_Ca_ channels, but its actions can enhance BK_Ca_ channel activity.

### 2.3. Stimulatory Effect of QO-40 on Large-Conductance Ca^2+^-Activated K^+^ (BK_Ca_) Channels in GH_3_ Cells

**Figure 4:**
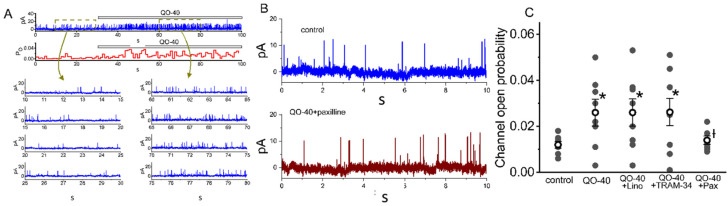
Stimulatory effect of QO-40 on the large-conductance Ca^2+^-activated K^+^ (BK_Ca_) channels identified in GH_3_ cells. The single-channel experiments in an excised inside-out membrane patch were undertaken with symmetrical K^+^ concentration (145 mM). The bath medium contained 0.1 μM Ca^2+^, and we kept the patch in voltage clamp at the level of +60 mV. (**A**) Representative current trace (upper, blue color) and the open probability (lower, red color) showing changes in the activity of BK_Ca_ channels after addition of QO-40 (3 μM). Channel openings are indicated as upward deflections, and the horizontal bar shown above either current tracings or time course of single open probability corresponds to the application of QO-40 to the bath. The lower parts in (**A**) depict expanded records obtained from the dashed boxes in the uppermost part. Current traces in the left or right side indicate the absence or presence of 3 μM QO-40, respectively. Note that the presence of QO-40 leads to an increase in channel open-state probability of BK_Ca_ channels. (**B**) BK_Ca_-channel activity obtained in the control period (i.e., neither QO-40 nor paxilline was present) (upper) and QO-40 (3 μM) plus paxilline (1 μM) (lower). In the experiments on QO-40 plus paxilline, paxilline (1 μM) was further added, as patch was continually exposed to QO-40 (3 μM). (**C**) Vertical scatter graph showing effects of QO-40, QO-40 plus TRAM-34, QO-40 plus linopirdine, or QO-40 plus paxilline on channel open-state probability of BK_Ca_ channels (mean ± SEM; *n* = 8 for each point). Lino: 10 μM linopirdine; TRAM-34: 3 μM TRAM-34; Pax: 1 μM paxilline. Data analysis was performed by ANOVA-1 (*p* < 0.05). * Significantly different from control (*p* < 0.05) and † significantly different from QO-40 (3 μM) alone group (*p* < 0.05).

As I_K(Ca)_ is biophysically a large, noisy, voltage-dependent, Ca^2+^-sensitive K^+^ current, its current strength is mostly due to the opening of BK_Ca_ channels in GH_3_ cells [17,18,26]. Therefore, we further explored whether QO-40 has any effect on the activity of BK_Ca_ channels. All inside-out current recordings are presented, and the following recordings were bathed in a symmetrical K^+^ concentration (145 mM) and bath medium containing 0.1 μM Ca^2+^. During measurements, we kept the cell in a voltage clamp at a holding potential of +60 mV. As illustrated in Figure 4A, after the addition of QO-40 (3 μM) into the cytosolic leaflet of the channel, a drastic increase in channel open-state probability was observed. As they were recorded from the detached patches of GH_3_ cells, the probabilities that BK_Ca_ channels would be open significantly and consistently increased from 0.013 ± 0.008 to 0.029 ± 0.014 (*n* = 8, *p* < 0.05) during exposure to 3 μM QO-40. After washout of the compound, the channel activity was reduced to 0.015 ± 0.008 (*n* = 7, *p* < 0.05). As shown in Figure 4B, BK_Ca_-channel activity did not differ between the absence (i.e., in the control period) and presence of of 3 μM QO-40 plus 1 μM paxilline. Moreover, in the continued presence of QO-40, neither linopirdine (10 μM) nor TRAM-34 (3 μM) could attenuate QO-40-mediated stimulation of BK_Ca_ channel activity, although further application of paxilline (1 μM) could reverse the increase in channel opening probability (Figure 4C).

### 2.4. Effect of QO-40 on Kinetic Behavior of BK_Ca_ Channels

**Figure 5:**
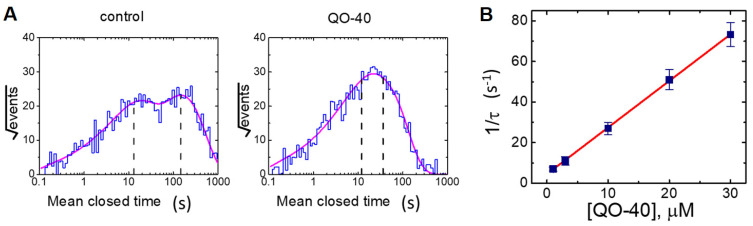
Effect of QO-40 on the mean closed time of BK_Ca_ channels identified in GH_3_ cells (**A**) and the relationship of the reciprocal of slow component in the mean closed time of the channel versus the QO-40 concentration (**B**). In (**A**), mean closed-time histogram of BK_Ca_ channels in the absence (left) or presence (right) of QO-40 (10 μM) in GH_3_ cells is illustrated, respectively. Under symmetrical K^+^ concentrations (145 mM) in which bath medium contained 0.1 μM Ca^2+^, the potential was voltage-clamped at +60 mV, and the inside-out configuration was performed. The closed-time histogram in the absence or presence of 10 μM QO-40 was least-squares fitted by a sum of two-exponential function (indicated by nonlinear continuous curve, pink color) with a mean closed time of 13 and 146 ms, or 12 and 36 ms, respectively. The x- or y-axis indicates the logarithm of mean closed time (ms) or the square root of the event number, respectively, and the broken line in each lifetime distribution is pointed at the values of the fast or slow component of time constant in closed (resting) states of the channel. Data in the control were obtained from a measurement of 232 channel openings with a total recording time of 1 min, whereas those in the presence of 10 μM QO-40 were from 439 channel openings with a total record time of 30 s. In (**B**), the reciprocal of slow component in the mean closed time of the channel (i.e., 1/t) versus the QO-40 concentration was derived and plotted. Data points indicated in filled squares were fitted by a linear regression (red color); hence, a molecularity of one was inferred. According to the first-order binding scheme elaborated in Section 4, forward (on, *k*_+1_^*^) or backward (off, *k*_−1_) rate constant for QO-40-induced decrease in the slow component of the mean closed time of the channel was calculated to be 2.298 s^−1^μM^−1^ or 4.512 s^−1^, respectively. Mean ± SEM (*n* = 8–10 for each point).

We continued to analyze the effects of QO-40 on the gating mechanisms that control the opening or closing of these channels due to its ineffectiveness in changing the single-channel amplitude. In excised patches of control cells (i.e., QO-40 was not present), open- or closed-time histograms at the level of +60 mV can be fitted with the goodness of fit by a one- or two-exponential curve, respectively. Of note, in detached patches, the addition of QO-40 (3 μM) did not change the mean open time of the channel (2.22 ± 0.12 ms (control) versus 2.24 ± 0.13 ms (in the presence of 3 μM QO-40); *n* = 7, *p* > 0.05). However, as demonstrated in Figure 5A, the slow component of mean closed time of BK_Ca_ channels was shortened to 36 ± 4 ms (*n* = 7, *p* < 0.05) from a control of 146 ± 11 ms (*n* = 7), although no modification in the fast component of mean close time of the channel was detected (13 ± 3 ms (control) versus 12 ± 3 ms (in the presence of 3 μM QO-40); *n* = 7, *p* > 0.05). Herein, the results showed that the presence of QO-40 could result in an evident decrease in the channel closure time, notwithstanding its ineffectiveness on the mean opening time of the channel observed at +60 mV (2.32 ± 0.12 ms (in control) versus 2.33 ± 0.13 ms (in the presence of QO-40); *n* = 7, *p* > 0.05). Due to the lack of change in single-channel amplitude, such perturbations in the gating closing of the channel help account for its stimulatory effect on BK_Ca_ channels present in GH_3_ cells.

In an effort to quantitatively estimate the QO-40-mediated decrease in the slow component of the mean closure time of BK_Ca_ channels recorded from GH_3_ cells, changes in the mean closure times at varying QO-40 concentrations were further analyzed. The concentration dependence of changes in the slow component of the mean closed time in the presence of QO-40 is illustrated in Figure 5B. The presence of QO-40 led to a concentration-dependent increase in the reciprocal (i.e., 1/τ) of the slow component in the mean closed time of the BK_Ca_ channel. A linear relationship between 1/τ and the QO-40 concentration with a correlation coefficient of 0.96 is illustrated (Figure 5B). The minimal reaction scheme detailed in Section 4 is closed ↔open ↔open·bound. The forward (or on) or backward (or off) rate constant was thereafter analyzed and yielded 2.298 s^−1^ μM^−1^ or 4.512 s^−1^, respectively; therefore, the value of the dissociation constant (*K*_D_ = *k*_−1_/*k*_+1_^*^) was 1.96 μM. Of note, this value is consistent with the effective EC_50_ of the QO-40-stimulated *I*_K(Ca)_ amplitude described above. Therefore, the QO-40-stimulated *I*_K(Ca)_ observed in whole-cell mode is greatly linked to its reduction in the mean closure time of BK_Ca_ channels.

### 2.5. Effect of QO-40 on the Steady-State Activation Curve of BK_Ca_ Channels in GH_3_ Cells

**Figure 6:**
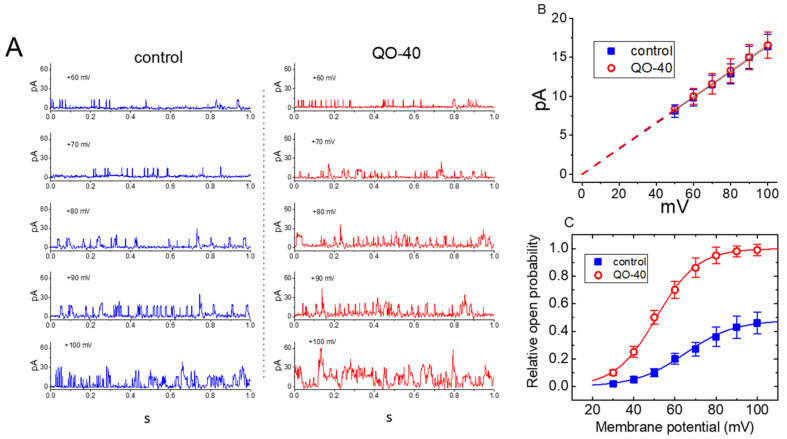
Effect of OD-40 on BK_Ca_-channel activity measured at the different levels of membrane potentials. Inside-out current recordings were performed in these experiments; cells were bathed in high-K^+^ solution (145 mM) containing 0.1 μM Ca^2+^. (**A**) Representative current traces obtained in the absence (left, blue color) and presence (right, red color) of 3 μM OD-40. The number shown in each panel indicates the membrane potential held, and the upper deflection is the opening event of the channel. (**B**) Relationship of single-channel current versus membrane potential (mean ± SEM; *n* = 9 for each point). The dashed lines were pointed to the reversal potential with 0 mV. Notice that the two lines are virtually superimposed, indicating the single-channel conductance of BK_Ca_ channels did not differ between the absence (filled symbol, blue color) and presence (open circles, red color) of OD-40. (**C**) The steady-state activation curve of BK_Ca_ obtained with or without addition of 3 μM OD-40 (mean ± SEM; *n*-7 for each point). The statistical analyses were undertaken by Student’s *t*-tests (*p* < 0.05). Continuous sigmoidal lines were best fit to the modified Boltzmann equation as described under Section 4.

**Figure 7:**
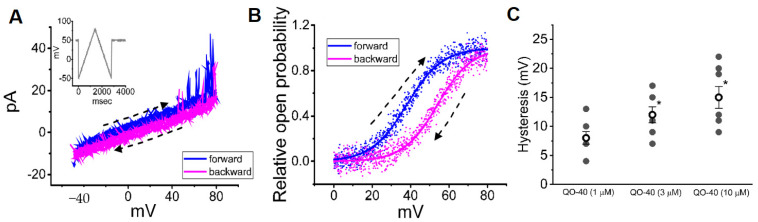
Effect of QO-40 on the voltage-dependent hysteresis of BK_Ca_ channels identified from GH_3_ cells. In this set of inside-out current recordings, we bathed cells in symmetrical K^+^ solution (145 mM). (**A**) Representative current traces obtained in the presence of QO-40 (3 μM). Channel activities were activated in response to long isosceles-triangular ramp pulse with a duration of 2.8 sec (indicated in the Inset). The dashed arrow indicates the direction of current flow through the channel in which time passes. (**B**) The relationship of the relative channel open probability versus membrane potential of BK_Ca_ channels in response to the forward (blue color) or backward (pink color) limb of triangular ramp pulse. (**C**) Vertical scatter graph showing effect of varying QO-40 concentrations on the hysteresis of BK_Ca_ channels. Hysteresis was measured at the voltage separation between the forward and backward limb at 50% of the relative channel open probability. Inside-out configuration was made, and a ramp speed of ±93 mV/s was applied to the patch. Each point indicates the mean ± SEM (*n* = 7). Data analysis was performed by ANOVA-1 (*p* < 0.05). * Significantly different from 1 μM QO-40 alone group (*p* < 0.05).

We continued to measure the amplitude of single BK_Ca_ channels at different voltages ranging between +50 and +100 mV. Throughout all voltage ranges examined, the I-V relationship of the BK_Ca_ channel in the absence or presence of QO-40 was compared. In the control (i.e., QO-40 was not present), fitting these current amplitudes with a linear regression revealed a single channel conductance of 162 ± 8 pS (*n* = 9). As illustrated in Figure 6A,B, the value did not differ significantly from that (164 ± 9 pS, *n* = 9) taken in the presence of QO-40 (3 μM). Therefore, when applied intracellularly, QO-40 does not appear to perturb the single-channel conductance of the channel, but it does greatly increase the activity of BK_Ca_ channels in GH_3_ cells. Figure 7C illustrates the steady-state activation curve of BK_Ca_ channels obtained in the absence or presence of QO-40 (3 μM). The relationship between the membrane potentials and the probabilities of BK_Ca_ channel openings with or without the addition of this compound (3 μM) was constructed and plotted. The data were least-squares fitted by the Boltzmann equation, as elaborated in Section 4. In the control (i.e., QO-40 was not present), *n* = 0.052 ± 0.009, V_1/2_ = 73.1 ± 2.6 mV, and q = 6.5 ± 0.8 e (*n* = 7), while in the presence of QO-40 (3 μM), *n* = 0.156 ± 0.011, V_1/2_ = 57.1 ± 7 mV, and q = 8.8 ± 0.8 e (*n* = 7). Therefore, the presence of QO-40 (3 μM) produced a 3-fold increase in the maximal open-state probability of BK_Ca_ channels, shifted the steady-state activation curve of a less positive membrane potential by 14 mV, and increased the gating charge by 1.4-fold. These results demonstrate that QO-40 can stimulate the activity of BK_Ca_ channels in a voltage-dependent manner in GH_3_ cells.

### 2.6. Effect of QO-40 on the Voltage-Dependent Hysteresis of BK-Channel Activity Elicited in Response to a Long-Lasting Isosceles-Triangular Ramp Pulse

The voltage-dependent hysteresis of ionic currents, namely, a lag in current magnitude as the linear voltage command is changed in the opposite direction, exerts noticeable actions reminiscent of electrical activity, such as action potential firing (i.e., initial depolarization and late repolarization) [27,28]. Therefore, we explored whether voltage-dependent hysteresis existed in the BK_Ca_ channel activity recorded from GH_3_ cells. In this set of inside-out experiments, we exploited a long (2.8 s in duration) upright triangular ramp pulse with a ramp speed of ±93 mV/sec for measurements of the hysteretic characteristics (Figure 7A). Of note, as illustrated in Figure 7, the trajectory of channel activity activated by the upsloping ramp pulse (i.e., the voltage change from −50 to +80 mV) and downsloping (i.e., the change from +80 to −50 mV) as a function of time was overly discriminated between these two limbs. In other words, in the presence of 3 μM QO-40, the relative channel open probability evoked by the upsloping (forward) end of the upright isosceles-triangular voltage ramp was higher than that in response to the downsloping (backward) end (Figure 7B). The observations enabled us to demonstrate that the voltage dependence of deactivation might shift to more positive potentials with increasing QO-40 concentration as more energy is required to close the channels and that the mode shift corresponds to the presence of distinct protein conformations [28,29].

For quantification, we further evaluated the degree of voltage-dependent hysteresis based on the voltage separation between the upsloping and downsloping branches at 50% of the relative channel open probability of BK_Ca_ channels [27]. As shown in Figure 7C, the presence of QO-40 can concentration-dependently increase the overall hysteretic strength of BK_Ca_ channels in GH_3_ cells. For example, as single-channel recordings were established, the addition of 3 or 10 μM QO-40 could increase the hysteretic strength up to 12.1 ± 1.4 or 14.9 ± 1.9 mV, respectively.

## 3. Discussion

In this study, QO-40 (3 μM) not only produced a shift of 14 mV to a less positive potential in the steady-state activation curve of BK_Ca_ cells but also increased the gating charge by 1.4-fold; however, it failed to change the single-channel conductance of the channel. The QO-40-mediated stimulation of BK_Ca_ channels in GH_3_ cells was not attenuated by linopirdine or TRAM-34 but was attenuated by paxilline. With the long-lasting isosceles-triangular ramp pulse, the presence of different QO-40 concentrations enhanced the voltage-dependent hysteretic strength of BK_Ca_ channels. Although the detailed mechanism of the stimulatory actions of QO-40 on BK_Ca_ channel activity is not yet known, experimental observations suggest that QO-40 can enhance the activity of BK_Ca_ channels in a voltage-dependent manner. Consequently, its interaction with the channel would considerably vary with either the pre-existing level of resting potential, the discharge patterns of action-potential firing, the concentration of QO-40 used, or any combinations.

In the present report, the effective EC_50_ value required for the QO-40-induced stimulation of macroscopic *I*_K(Ca)_ seen in GH_3_ cells was estimated to be 2.3 μM, a value that is lower than the EC_50_ (3.5 μM) needed for cilostazol-stimulated BK_Ca_ channels. Cilostazol was previously reported to be a stimulator of BK_Ca_ channels [29]. Moreover, according to the first-order reaction scheme stated in Section 4, we were able to yield the *K*_D_ value needed for its shortening in the slow component of the mean closure time of the BK_Ca_ channel with 1.96 μM, a value that is close to the EC_50_ value for QO-40-stimulated *I*_K(Ca)_. These results demonstrate that QO-40-stimulated *I*_K(Ca)_ in GH_3_ cells is largely accounted for by its decrease in the mean closure time of BK_Ca_ channels. Alternatively, the maximal concentration of QO-58, a synthesized compound that is structurally similar to QO-40, following oral administration at 25, 50, or 100 mg/kg, has been previously reported to reach 8.25, 16.29 or 18.27 mg/liter (i.e., approximately 18.6, 37, or 41 μM), respectively [30]. In this scenario, the stimulatory effects of QO-40 on BK_Ca_ channels demonstrated here would apparently be of pharmacological or therapeutic value [3], as this compound at lower concentrations is effective at stimulating *I*_K(Ca)_ and enhancing BK_Ca_-channel activity. Indeed, the QO-40 concentrations for these actions tend to overlap those needed for the activation of KCNQ currents [1,3,16]. The compounds structurally similar to QO-40 (i.e., PPO derivatives with different groups substituted at the C-2, C-3, and C-5 positions), as reported previously [1,16], are likely to elicit similar results as QO-40′s interaction with BK_Ca_ channels, which can increase the channel open-state probability and consequently enhance whole-cell *I*_K(Ca)_ amplitude. Due to their high potency, the emerging data may be important in interpreting the in vivo mechanism of actions of this compound and its structurally similar PPOs. Alternatively, it remains to be answered whether the rank potency order for these synthetic PPO derivatives in activating BK_Ca_ channels would share a similar magnitude for their stimulation of KCNQ currents.

Of note, the M-type K^+^ current (*I*_K(M)_) activated by membrane depolarization may somehow coincide with other types of outward K^+^ currents (e.g., *I*_K(Ca)_) existing in different types of cells, including CHO or HEK293 native cells. Neurons derived from dorsal root ganglia have abundant BKCa channel activity, which is thought to be closely linked to pain sensation [2,8,11,31,32,33]. As such, the ameliorating effects of QO-40 on inflammatory pain in rodents, as reported previously [3,5], could be partly, if not entirely, explained by its concurrent and synergistic activation of BK_Ca_ channels, which remain functionally active in dorsal root ganglion neurons [8].

The results from the inside-out current recordings are important because they suggest that QO-40 may bind to a site located in the cytoplasmic leaflet of the α-subunit, thereby resulting in the elicitation of BK_Ca_ channels in a concentration-, voltage-, and state-dependent manner. It is reasonable to speculate, therefore, that in addition to KCNQ channels [1,30], the BK_Ca_ channel α-subunit may be another important target of QO-40 on plasmalemmal ion channels. However, the extent to which QO-40 or other PPO derivatives affect other variants of BK_Ca_ channels in different types of cells needs to be further investigated. BMS-204352 or naringenin is an activator of both BK_Ca_ channels and KCNQ-encoded K^+^ currents [15]. It is thus plausible to note from the present observations that BK_Ca_ and KCNQ2/KCNQ channels share unique motifs or recognition sequences with which some small-molecule compounds can interact.

The activation of BK_Ca_ channels induced by QO-40 in GH_3_ cells also shared similar characteristics as those demonstrated in HEK293T cells in which *α-hSlo* channels were functionally expressed (Supplementary Information Figure S1). Our results suggest that QO-40 may bind to a site located on the cytoplasmic side of the α-subunit. However, it remains to be resolved whether different accessory β-subunits of the channel may affect the QO-40-stimulated activity of BK_Ca_ channels.

An equally important finding observed in this study was the emergence of voltage-dependent hysteresis of single BK_Ca_ channel activity activated in response to the long upright isosceles-triangular ramp pulse when the detached patches of GH_3_ cells were exposed to varying QO-40 concentrations. With increasing QO-40 concentration, the hysteretic strength of the channel (i.e., difference in the voltage between the upsloping and downsloping limbs at 50% channel open probability) was robustly enhanced, suggesting that as the QO-40 concentration increased, deactivation of the channel intrinsically changed the voltage dependence in situations where the V_1/2_ values at the forward and backward ends of the triangular ramp widened. However, in the presence of varying QO-40 concentrations, no obvious change in the single-channel conductance of BK_Ca_ channels during the hysteretic changes activated in response to a long triangular ramp pulse was demonstrated, although the channel open probability existing at the forward and backward limbs of the isosceles-triangular ramp pulse became overly distinguishable. Therefore, the observed change in voltage-dependent hysteresis in the presence of QO-40 does not appear to be located at the pore region of the channel, despite being intrinsically hysteretic in channel activity. Nevertheless, either the voltage dependence of BK_Ca_ channels or a significant mode shift, where there is voltage sensitivity in the gating charge movements of the current, might potentially appear in the presence of QO-40 [27,28]. Further work is required to evaluate the extent to which the direct QO-induced hysteretic changes (i.e., dynamic voltage dependence) in BK_Ca_ channels impact cell behavior in different cell types.

Some compounds known to augment *I*_K(M)_ (e.g., diclofenac, naringenin, or flupirtine) have previously been shown to perturb other types of voltage-activated K^+^ currents [15,34,35]. It appears that QO-40 or other structurally similar compounds (e.g., PPO derivatives) do not exclusively act on KCNQ channels, as suggested previously [1,3,16]. From this perspective, QO-40′s modifications of ionic currents described presently with effective EC_50_ or *K*_D_ values required for the stimulation of BK_Ca_ channels may well be repurposed or entailed to exert a strong impact on the functional activities of varying cell types occurring in vitro or in vivo if they functionally express BK_Ca_ channels. Regardless of the mechanisms possibly involved, the effectiveness of QO-40 or other structurally similar compounds in activating BK_Ca_ channels should be noted carefully in relation to its increasing use as an activator of KCNQ/M channels.

Of note, the *I*_K(Ca)_ found in glioma cells are mediated by an isoform of the BK_Ca_ channel, termed gBK, which contains a 34-amino-acid insert at splice sites [36]. Additionally, the mitochondrial BK_Ca_ channels have been previously reported [37]. Whether QO-40 can exert any effects on these glial or mitochondrial BK_Ca_ channels or on other channel proteins remains to be further investigated.

The study limitation in this study must be emphasized. In the whole cell experiments, QO-40 was applied extracellularly, while in inside-out current recordings, it was applied intracellularly. In whole-cell mode, current amplitudes were contaminated with Ca^2+^ ions and 0.1 mM EGTA, while in single-channel recordings, 0.1 μM Ca^2+^ was present in bath medium. Moreover, the voltage ranges applied to activate BK_Ca_ in this study were observed to be more positive, although this could be due to the possibility that the bath medium contained high-K^+^ solution. Therefore, the experimental conditions in this study might not be physiological.

## 4. Materials and Methods

### 4.1. Chemicals and Solutions Used in This Work

QO-40 (5-(chloromethyl)-3-(naphthalen-1-yl)-2-(trifluoromethyl)pyrazolo[1,5-a]pyrimidin-7(4*H*)-one, 5-(chloromethyl)-3-naphthalen-1-yl-2-(trifluoromethyl)-1H-pyrazolo[1,5-a]pyrimidin-7-one, C_18_H_11_ClF_3_N_3_O, https://www.alomone.com/p/qo-40/Q-265, https://pubchem.ncbi.nlm.nih.gov/#query=QO-40, accessed on 16 May 2011), and paxilline were acquired from Alomone (Asia Bioscience, Taipei, Taiwan). GAL-021 was obtained from MedChemExpress (Biogenesis Technologies, Taipei, Taiwan), linopirdine was obtained from Sigma-Aldrich (Merck, Taipei, Taiwan), and TRAM-34 was obtained from Togenesis Technologies (Taipei, Taiwan). QO-40 was dissolved in DMSO as a 20 mM stock solution, and it was thereafter diluted in extracellular solution to the final concentrations achieved; the vehicle at the final concentration did not affect the channel activity. QO-40 stock solutions were refrigerated at 4 °C and kept in foil to prevent light degradation. For cell preparations, we acquired all culture media, fetal calf serum, horse serum, L-glutamine, and trypsin/EDTA from HyClone^TM^ (Thermo Fisher; Level Biotech, Tainan, Taiwan), whereas other chemicals were of analytical grade. In the experiments, we used double-distilled water that had been deionized through a Millipore-Q purification system (Merck, Taipei, Taiwan).

The composition of extracellular solution (i.e., HEPES-buffered normal Tyrode’s solution) was as follows (in mM): NaCl 136.5, KCl 5.4, CaCl_2_ 1.8, MgCl_2_ 0.53, glucose 5.5, and HEPES 5.5 adjusted to pH 7.4 with NaOH. To measure flow through macroscopic K^+^ currents (e.g., *I*_K(Ca)_), we filled a pipet with the following solution (in mM): KCl 140, MgCl_2_ 1, Na_2_ATP 3, Na_2_GTP 0.1, EGTA 0.1, and HEPES 5 adjusted to pH 7.2 with KOH. To record the activity of BK_Ca_ channels under the inside-out configuration, the bath solution contained a high K^+^ solution (in mM): KCl 130, NaCl 10, MgCl_2_ 3, glucose 6, and HEPES-KOH buffer 10 adjusted to pH 7.4 with KOH. Based on a dissociation constant of 0.1 μM for EGTA and Ca^2+^ (at pH 7.2), we estimated the free Ca^2+^ concentration. For example, to provide 0.1 μM Ca^2+^, we added 1 mM EGTA, and 0.5 mM CaCl_2_ was added to the bath solution. We also filtered the pipet solutions and culture media on the day of measurements with an Acrodisc^®^ syringe filter with a 0.2 μm Supor^®^ membrane (Bio-Check; New Taipei City, Taiwan).

### 4.2. Cell Preparations

The GH_3_ clonal cell line was acquired from the Bioresources Collection and Research Center ([BCRC-60015]; Hsinchu, Taiwan). We cultured cells in Ham’s F-12 medium supplemented with 15% (*v*/*v*) horse serum, 2.5% (*v*/*v*) fetal calf serum, and 2 mM l-glutamine. To promote cell differentiation, we transferred cells to serum-free, Ca^2+^-free medium. Cells were maintained at 37 °C in a humidified atmosphere containing 95% air and 5% CO_2_. Cell viability was evaluated using the trypan blue dye exclusion test. For subculturing, we trypsin-dissociated cells and passaged them every 2–3 days, while a new stock line was generated from frozen cells (frozen in 10% glycerol in medium plus serum) every 3 months. Electrical recordings were performed five or six days after cells had been cultured (60–80% confluence). The preparation of *α-hSlo*-expressing HEK293T cells is described in the Supplementary Information Figure S1.

### 4.3. Electrophysiological Recordings

Immediately before each experiment, GH_3_ or HEK293T cells were dispersed, and an aliquot of cell suspension was rapidly transferred to a custom-built recording chamber and allowed to settle to the bottom of the chamber. The recording chamber was firmly positioned on the stage of an inverted phase-contrast microscope (Diaphot-200; Nikon; Lin Trading Co., Taipei, Taiwan). The microscope was coupled to a video camera system with magnification up to 1500× to monitor cell size during the experiments. Cells were kept in a bath at room temperature (20–25 °C) in normal Tyrode’s solution containing 1.8 mM CaCl_2_. The patch pipets were pulled from Kimax-51 thin-walled unfilamented capillaries with a 1.5–1.8 mm outer diameter (#34500; Kimble; Dogger, New Taipei City, Taiwan) using a two-stage PP-83 puller (Narishige; Taiwan Instrument, Tainan, Taiwan), and the tips were then fire-polished with an MF-83 microforge (Narishige). When filled with pipet solution, the resistances ranged between 3 and 5 MΩ. We performed standard patch-clamp recordings in cell-attached, inside-out, or whole-cell configurations using an RK-400 amplifier (Bio-Logic, Claix, France) [17,18]. Any seals less than 1 GΩ were discarded. Junctional potentials, which develop at the pipet tip when the composition of the internal solution differed from that in the bath, were nulled before the start of each giga-seal formation, and such potentials then corrected the whole-cell data. The tested compounds were either applied through perfusion or added to the bath to achieve the final concentration indicated. During measurements, the signals, consisting of voltage and current tracings, were stored online at 10 kHz in an ASUSPRO-BU401 LG laptop computer (ASUS, Tainan, Taiwan) equipped with a Digidata-1440A device (Molecular Devices; Advance Biotech, New Taipei City, Taiwan) and controlled by pCLAMP 10.7 software (Molecular Devices).

### 4.4. Data Analyses

To calculate the percentage stimulation of *I*_K(Ca) by QO-40_, we incubated cells in normal Tyrode’s solution containing 1.8 mM CaCl_2_. The examined cell was 300 ms depolarized from 0 to +50 mV at a rate of 0.1 Hz, and the *I*_K(Ca)_ amplitude taken at varying QO-40 concentrations was measured at the end of the depolarizing pulse. The amplitude of *I*_K(Ca)_ in the presence of 100 μM QO-40 was taken as 100%, and those achieved during exposure to different QO-40 concentrations (0.3–30 μM) were thereafter compared. We determined the concentration–response relationship of QO-40-stimulated *I*_K(Ca)_ amplitude in GH_3_ cells by using the least-squares fitting of data to the Hill equation:

$$\%\;\mathrm{increase}=\frac{{\left[\mathrm{QO}-40\right]}^{n_{H}}\times E_{max}}{{\left[\mathrm{QO}-40\right]}^{n_{H}}+EC_{50}^{n_{H}}}$$

where *EC*_50_ or *n_H_* is the half-maximal concentration of QO-40 or the Hill coefficient, respectively; [QO-40] is the QO-40 concentration; and *E_max_* is the maximal activation of *I*_K(Ca)_ caused by this compound.

### 4.5. Single-Channel Analyses

Single BK_Ca_ channel amplitudes identified in GH_3_ or HEK-293T cells were determined by fitting Gaussian distributions to the amplitude histograms of the closed (resting) and open states. The probabilities of channel openings in a patch were expressed as *N*·*P*_O_, which was estimated using the following equation:

$$N\cdot P_{O}=\frac{\left(A_{1}+2A_{2}+\ldots +nA_{n}\right)}{\left(A_{0}+A_{1}+A_{2}+\ldots +A_{n}\right)}$$

where *A* indicates the area under the curve of an all-points histogram that corresponds to the closed (resting) state, *A*_1_…*A_n_* are the histogram areas indicating the levels of distinct open state for 1 to *n* channels in the patch, and *N* represents the number of active channels in the patch. Open- or closed-time distributions with or without the addition of QO-40 were fitted with logarithmically scaled bin width. For dwell-time analyses, only one single channel in the patch was used.

The stimulatory effect of QO-40 on BK_Ca_ channel activity is thought to be ascribed to a state-dependent stimulator that binds predominantly to the closed (or resting) state of the channel. Based on this simplifying assumption, a minimal reaction scheme was given as follows:

$$C\;\overset{\alpha }{\underset{\beta }{⇄}}\;O\;\overset{{k_{+1}}^{*}\cdot [\mathrm{QO}-40]}{\underset{k_{-1}}{⇄}}\;O\cdot [\mathrm{QO}-40]$$

or,

$$\frac{dC}{dt}=O\times \beta -C\times \alpha$$

$$\frac{dO}{dt}=C\times \alpha +O\cdot \left[\mathrm{QO}-40\right]\times k_{-1}-O\times \beta -O\times k_{+1}^{*}\cdot \left[\mathrm{QO}-40\right]$$

$$\frac{d\left(O\cdot \left[\mathrm{QO}-40\right]\right)}{dt}=O\times k_{+1}^{*}\cdot \left[\mathrm{QO}-40\right]-O\cdot \left[\mathrm{QO}-40\right]\times k_{-1}$$

where [QO-40] is the QO-40 concentration used, and *α* or *β* is the voltage-gated rate constant for the opening or closing of the BK_Ca_ channel, respectively. *k*_+1_^*^ or *k*_−1_ represents the forward (i.e., on or bound) or backward (i.e., off or unbound) rate constant of QO-40, respectively, whereas *C*, *O*, or *O*·[QO-40] in each term represents the closed (resting), open, or open-[QO-40] state, respectively.

The value of k^*^_+1_ or *k*_−1_ was evaluated based on the mean closed time in the slow component of BK_Ca_ channels attained during exposure to varying QO-40 concentrations. Using the above-described binding scheme, these rate constants could be optimized using the following equation:

$$\frac{1}{\tau }=\left[\mathrm{QO}-40\right]\times k_{+1}^{*}+k_{-1}$$

where k^*^_+1_ or *k*_−1_ can be derived from the slope or from the y-axis intercept at [QO-40] = 0 of the linear regression, which interpolates the reciprocal time constants (i.e., 1/τ) versus the QO-40 concentration used, and [QO-40] is the QO-40 concentration. A measure of the dissociation constant (*K*_D_) equal to the *k*_−1_ value divided by the value of [KO-40]·k^*^_+1_ can thereafter be appropriately yielded.

The relationship between the membrane potentials and relative open-state probability of BK_Ca_ channels (i.e., the steady-state activation curve) with or without the application of QO-40 (10 μM) was constructed and fitted by the Boltzmann equation (or the Fermi-Dirac distribution) using the goodness-of-fit test [38]:

$$\mathrm{relative}\;N\cdot P_{O}=\frac{n}{\left\{1+e^{\left[\frac{-\left(V-V_{\frac{1}{2}}\right)\mathrm{qF}}{\mathrm{RT}}\right]}\right\}}$$

where N is the number of channels in the patch; n is the maximal relative N·P_O_; V is the membrane voltage in mV; V_1/2_ and q represent the potential for half-maximal activation and the apparent gating charge, respectively; and F, R, and T are Faraday’s constant, the universal gas constant, and the absolute temperature, respectively.

To determine the effect of QO-40 on the hysteretic strength of BK_Ca_ channels, a 2.8-sec upright triangular ramp pulse from −50 to +80 mV with a ramp speed of ±93 mV/s at a rate of 0.1 Hz was utilized and then applied to the detached patch with digital-to-analog conversion. To obtain the relative probabilities of channel openings in varying QO-40 concentrations, single-channel amplitudes in response to 20-voltage ramps were averaged, and each point of the averaged current was divided by the single-channel amplitude of each potential after the leak component was corrected [39]. The number of active channels in the patch, N, was also counted at the end of the experiments through the addition of a solution with 100 μM Ca^2+^ and then used to normalize the open-state probability. To obtain values for the gating charge and half-maximal activation of voltage, the curve obtained at the upsloping (forward) or downsloping (backward) limb of the triangular ramp pulse was fitted with Boltzmann functions as described above.

### 4.6. Statistical Analyses

Linear or nonlinear curve fitting of experimental data sets was achieved with the least-squares minimization procedure using various maneuvers, such as Microsoft Excel-embedded “Solver” (Microsoft, Redmond, WA, USA) and 64-bit OriginPro^®^ program (OriginLab; Scientific Formosa, Kaohsiung, Taiwan). The macroscopic or single-channel data are presented as the mean ± standard error of the mean (SEM) with sample sizes (n) indicative of the cell number from which the experimental results were achieved. We initially applied Student’s *t*-tests (paired or unpaired) for statistical analyses. Additionally, we performed either analysis of variance (ANOVA)-1 or ANOVA-2 with or without repeated measures followed by post hoc Fisher’s least-significant difference test. *p* values < 0.05 were considered significant.

## 5. Conclusions

The principal findings from this study are as follows: (a) QO-40 can concentration-dependently stimulate the amplitude of I_K(Ca)_; (b) QO-40-stimulated I_K(Ca)_ can be effectively reversed by either GAL-021 or paxilline, but not by linopirdine or TRAM-34; (c) QO-40 increases the probabilities of BK_Ca_ channels that would be open, although it did not change single-channel conductance; (d) QO-40-stimulated I_K(Ca)_ can be largely explained by a concentration-dependent reduction in the slow component of the mean closed time of the channel; (e) QO-40 produces a left shift in the steady-state activation curve of BK_Ca_ channels and increases the gating charge of the channel; and (f) this compound can enhance the hysteretic strength of BK_Ca_ channels elicited by the long isosceles-triangular ramp pulse. Taken together, apart from the well-established activation of KCNQ currents [1,16], the QO-40-mediated stimulation of BK_Ca_ channels described herein could highlight another yet unidentified but noticeable ionic mechanism of the actions produced by it or other structurally similar compounds (i.e., pyrazolol[1,5-a]pyrimidin-7(4H)-one [PPO] derivatives) through which they act on the functional activities of various cell types in vivo.

## Data Availability

The data presented in this study are available on request from the corresponding author.

## Supplementary Materials

The following are available online at https://www.mdpi.com/article/10.3390/ph14050388/s1, Figure S1: Stimulatory effect of QO-40 on BKCa-channel activity measured from α-hSlo-expressing HEK293T cells.

## Abbreviations

BK_Ca_ channel, big-(large- or high-) conductance Ca^2+^-activated K^+^ channel; EC_50_, concentration required for half-maximal stimulation; *I-V*, current versus voltage; *I*_K(Ca)_, Ca^2+^-activated K^+^ current; IK_Ca_ channel, intermediate-conductance Ca^2+^-activated K^+^ channel; *I*_K(M)_, M-type K^+^ current; *K*_D_, dissociation constant; PPO, pyrazolol[1,5-a]pyrimidin-7(4*H*)-one; QO-40, 5-(chloromethyl)-3-(naphthalene-1-yl)-2-(triflouoromethyl)pyrazolo[1,5-a]pyrimidin-7-(4*H*)-one; SEM, standard error of the mean.
