# Supplementary material for: Effective Activation of BKCa Channels by QO-40 (5-(Chloromethyl)-3-(Naphthalen-1-yl)-2-(Trifluoromethyl)Pyrazolo [1,5-a]pyrimidin-7(4H)-one), Known to Be an Opener of KCNQ2/Q3 Channels
Source: Pharmaceuticals (Basel). 2021 Apr 21;14(5):388. doi: 10.3390/ph14050388 (PMC8143083; doi:10.3390/ph14050388)
Supplement: Supplementary file 1 [file pharmaceuticals-14-00388-s001.zip › pharmaceuticals-1179848-supplementary.pdf]

Supplementary Information

Materials and Methods

Cell preparation

The HEK293T cell line was acquired from the American Type Culture Collection (CRL-11268; Manassas, VA, USA). Cells were grown in DMEM supplemented with 10% fetal bovine serum and 2 mM L-glutamine at 37 °C in an atmosphere of 5% CO_2 and 95% air incubator. For transfection of HEK293T cells, cells at a number of 0.8-2.4 × 10^5 were seeded on the 35-mm culture plate for 24 hours.

Transfection

The pCMV6-XL4 vector which contained human BK_{Ca}-channel pore-forming -subunit cDNA (-*hSlo*; NM_0022471) was acquired from Origen Technologies (Rockville, MD, USA). The -*hSlo* gene is recognized to encode a functional BK_{Ca} channel. The expression plasmid was transfected into HEK293T cells for transient expression (So et al., 2011). Briefly, the expression plasmid was prepared in 150 mM NaCl as a diluent solution. PEI (ExGen 500; MBI Fermentas, Hanover, MD, USA) and plasmid were mixed together and thereafter incubated for 10 min at room temperature for sufficient binding of the plasmid to PEI. We then added plasmid-PEI mixture solution to the 24-well plate and centrifuge it at 280 *g* for 5 min. After centrifugation, transfected cells were incubated at 37 °C for additional 48 hours. The expression of -*hSlo* channels was determined by either immunofluorescence staining or electrophysiological measurements.

Data analysis

To determine the percentage stimulation of QO-40 on the probability of BK_{Ca} channels that would be open, we kept HEK293T cells to be bathed in high-K^+ (145 mM) solution containing 0.1 M Ca^{2+}. In inside-out current recordings, the examined cell was voltage-clamped at +60 mV, and channel open probabilities taken at varying QO-40 concentrations were measured. The channel activity in the presence of 100 M QO-40 was taken as 100%, and those during exposure to different QO-40 concentrations were then compared. We then evaluated the concentration-response relationship of QO-40-induced increase of BK_{Ca}-channel activity in -*hSlo*-expressing HEK293T cells by virtue of least-squares-fitting of data to the Hill equations:

$$\%\ \text{increase} = \frac{[\text{QO-40}]^{n_H} \times E_{max}}{[\text{QO-40}]^{n_H} + EC_{50}^{n_H}}.$$

In this equation, EC_{50} or n_H is the half-maximal concentration of QO-40 or the Hill coefficient, respectively, [QO-40] the QO-40 concentration applied, and E_{max} the maximal increase of BK_{Ca} channels caused by this compound.

Results

Effect of QO-40 and QO-40 plus paxilline on the probability of BK_{Ca} channels that would be open in HEK293T cells transfected with a-hSlo.

The principal work in this study has demonstrated the effectiveness of QO-40 in suppressing the

activity of BK_{Ca} channels in pituitary GH_3 cells. We hence extended to test the hypothesis that this compound exercises any effects on BK_{Ca} channels in HEK293T cells expressing -*hSlo*. Under our experimental conditions, we were able to transfect cells with -*hSlo* and the appearance of BK_{Ca} channels was then noticed. As illustrated in **Supplementary Figures 1A and 1B**, in inside-out current recordings, QO-40 at a concentration of 3 M increased channel open probability at +60 mV from 0.020 ± 0.0015 to 0.0302 ± 0.0017 (n=6, P<0.05). As the patch was continually exposed to QO-40 (3 M), the further addition of paxilline (1 M) was noticed to reverse QO-40-stimulated channel activity, as demonstrated by a reduction of channel open probability to 0.0205 ± 0.0017 (n=6, P<0.05).

Concentration-dependent relation of QO-40-mediated raise in BK_{Ca}-channel activity observed in HEK293T cells transfect with -*hSlo* was constructed and is hence illustrated in **Supplementary Figure 1C**. As the data were analyzed, the results yielded an EC_{50} value of 2.1 M. Therefore, in agreement with the observations described in GH_3 cells, the QO-40-mediated stimulation of BK_{Ca}-channel activity in HEK293T cells which were transfected with -*hSlo*, is most likely to be associated with its increase in the probability of channel openings, rather than changes in the number of functional active channels. Moreover, QO-40 was able to interact with BK_{Ca} channels in these cells which are devoid of BK_{Ca}-channel -subunits.

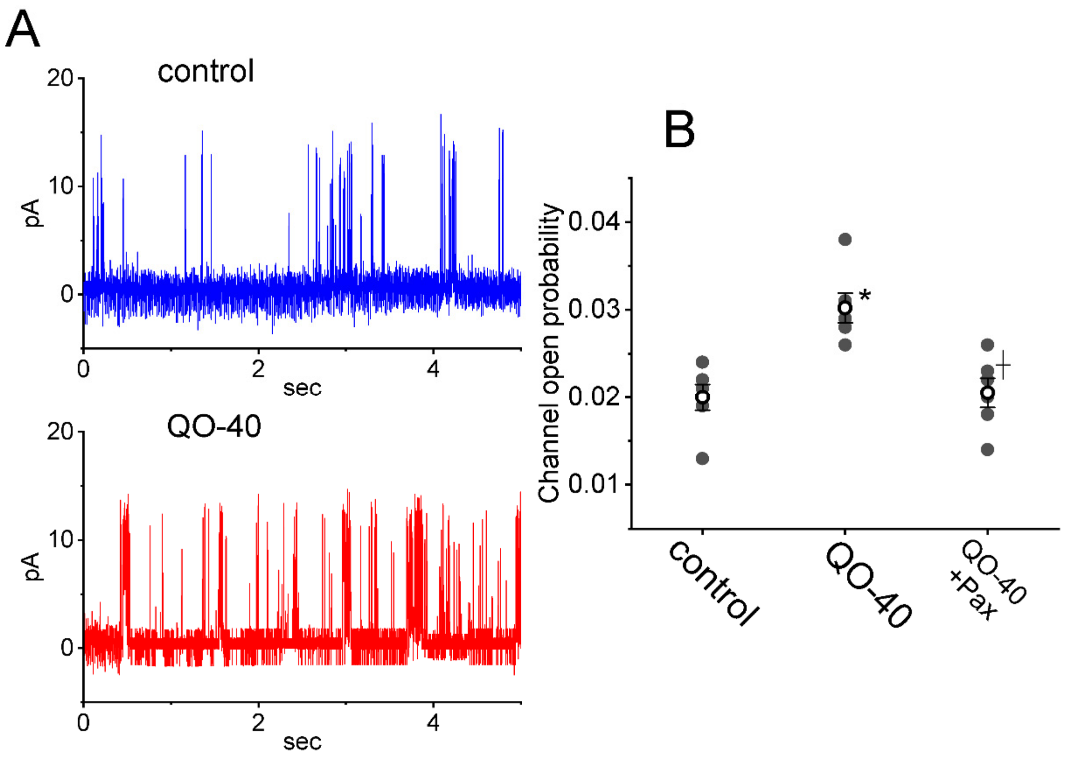

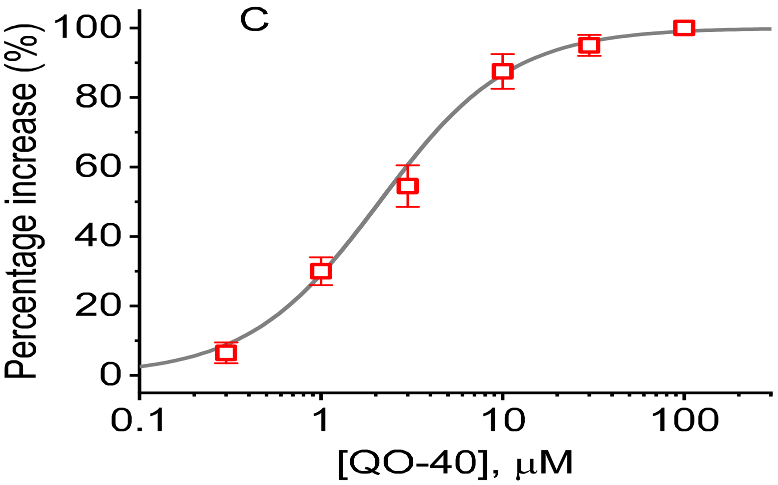

Supplementary Figure S1. Stimulatory effect of QO-40 on BK_{Ca}-channel activity measured from -*hSlo*-expressing HEK293T cells. **(A)** Representative current traces demonstrating the activity of BK_{Ca} channels before (upper, blue color) and after the application (lower, red color) of 3 M QO-40. Inside-out current recordings were undertaken with symmetrical K^+ concentration (145 mM). We voltage-clamped the patch at a potential of +60 mV, and bath medium contained 0.1 M Ca^{2+}. Channels openings show an upward deflection in current. **(B)** Vertical scatter graph showing effect of QO-40 (3 M) and QO-40 (3 M) plus paxilline (Pax; 1 M) on the probability of BK_{Ca}-channel openings (mean ± SEM; *n* = 6 for each point). *, Significantly different from control (i.e., QO-40 was not present) (P<0.05) and ⁺, significantly different from QO-40 (3 M) alone group (P<0.05). **(C)** Concentration-response relationship for QO-40-induced increase of BK_{Ca} channels that would be open. The smooth gray line with which the data points were overlaid is well fitted to the Hill equation. The values for EC_{50}, maximal percentage increase of channel open probability, and the Hill coefficient were 2.1 M, 100%, and 1.2, respectively. Each point represents the mean ± SEM (*n* = 7).

Discussion

In our study, control trace obtained in -*hSo*-expressing HEK293T cells tended to display a relatively low open probability at +60 mV. The reason could be presently unclear; however, it could be due to several reasons: First, a lack of accessory -subunit(s) in the channel was present in these cells. Second, the Ca^{2+} concentration in bath medium is relatively low (i.e. 0.1 M). Third, the experiments were conducted in a high-K^+ concentration (145 mM) with a reversal potential of around 0 mV; hence, the potential held at +60 mV could not be high enough to display high activity of BK_{Ca} channels, although high K^+ solution might increase the conductance of BK_{Ca} channels.

Moreover, we further examined the concentration-response relationship of QO-40-induced increase in BK_{Ca}-channel activity in -*hSlo*-expressing HEK293T cells. As the detached patches of these cells were exposed to different QO-40 concentrations, the effective EC_{50} value required for its increase in channel open probability was estimated to be 2.1 M (**Supplementary Figure 1C**), a value that is similar to EC_{50} for the stimulation of whole-cell $I_{K(Ca)}$ in GH_3 cells.

Reference

So EC, Wu KC, Liang CH, Chen JY, Wu SN. Evidence for activation of BK_{Ca} channels by a known inhibitor of focal adhesion kinase, PF573228. Life Sci 2011;89:691-701.
